# Culturally Competent Oncology Care for Muslim Patients: A Case-Based Narrative Review of Barriers and Clinical Outcomes

**DOI:** 10.7759/cureus.105396

**Published:** 2026-03-17

**Authors:** Omar Alkhabbaz, Asim Mohamed, Ahmed Alshaikhsalama, Shifa Kanjwal, Anas Alkhani, Bassel Atassi, Zahid Ahmad, Anas Alomar, Ayman Hallab, Luma Ghalib, Asrar Alahmadi, Rania Droubi, Hesham Mourad, Sawsan Rashdan

**Affiliations:** 1 Texas College of Osteopathic Medicine, University of North Texas Health Science Center, Fort Worth, USA; 2 Internal Medicine, University of Texas Southwestern Medical Center, Dallas, USA; 3 Ophthalmology, University of Texas Southwestern Medical Center, Dallas, USA; 4 Hematology and Oncology, University of Texas Southwestern Medical Center, Dallas, USA; 5 Internal Medicine, Northside Hospital Gwinnett, Lawrenceville, USA; 6 Hematology and Oncology, Little Company of Mary Medical Center, Evergreen Park, USA; 7 Endocrinology, University of Texas Southwestern Medical Center, Dallas, USA; 8 Interventional Cardiology, Methodist Health System, Dallas, USA; 9 Nephrology, Indiana University Health Bloomington Hospital, Bloomington, USA; 10 Endocrinology and Diabetes, The Ohio State University Wexner Medical Center, Columbus, USA; 11 Medical Oncology, The Ohio State University Wexner Medical Center, Columbus, USA; 12 Healthcare Administration, University of Houston - Clear Lake, Houston, USA; 13 Pharmacology, Mayo Clinic, Rochester, USA

**Keywords:** case-based narrative review, cultural considerations, cultural medicine, gender-sensitive care, muslim population, oncology care, patient centered care, spiritual care

## Abstract

Given the growing Muslim population in the United States, oncology providers increasingly encounter patients whose healthcare decisions are deeply influenced by religious and cultural beliefs. This paper addresses existing gaps in culturally competent cancer care for Muslim patients, identifies barriers affecting healthcare utilization, and proposes practical solutions to enhance clinical outcomes, patient trust, and patient satisfaction.

A case-based narrative review analyzes critical intersections between Islamic faith, cultural practices, and oncology care delivery. The case of a Muslim Syrian refugee woman with metastatic breast cancer is utilized to highlight real-world challenges related to delayed care-seeking behaviors, modesty and gender sensitivity, dietary restrictions, spiritual care needs, and end-of-life considerations. This analysis integrates findings from existing literature on health disparities, cultural competence frameworks, and ethical considerations relevant to oncology practice.

Muslim patients frequently encounter unique barriers to timely cancer diagnosis and treatment, such as spiritual interpretations of illness, modesty-related concerns, adherence to dietary laws, and misconceptions about palliative care. These factors contribute to reduced participation in cancer screening and delayed care, adversely impacting clinical outcomes. In the featured case, personalized interventions - including care provided by a female Muslim oncologist, integration of spiritual practices, and respectful accommodation of Islamic values - markedly improved patient engagement, treatment acceptance, and overall satisfaction.

To effectively serve Muslim oncology patients, healthcare systems and providers must embrace a culturally competent model of care. Training clinicians in cultural humility and Islamic healthcare considerations, implementing gender-sensitive protocols, ensuring access to halal dietary options, and integrating spiritual support can reduce healthcare disparities. An "Ask, Don't Judge" approach fosters patient-provider trust, ultimately promoting better clinical outcomes and enhancing patient experiences within oncology practice.

## Introduction and background

Introduction

Muslims are the fastest-growing faith group globally, with approximately 1.8 billion adherents as of 2020, comprising around 24% of the global population [1]. In the United States, there are an estimated 3.45 million Muslims, accounting for 1.1% of the population. Reports by the Pew Research Center project Muslims to be the second-largest faith group in the U.S. by 2050 [2]. Importantly, American Muslims are not exclusively immigrants from the Middle East or Asia; a substantial number are African Americans with a deep-rooted history in the U.S. [3]. This diversity within the American Muslim community underscores the need for healthcare providers to provide culturally competent care that respects patient autonomy, promotes beneficence, avoids harm, and ensures equitable treatment. It's also crucial to recognize that some Muslim families have been in the U.S. since its founding, while others are more recent immigrants, refugees, or asylum seekers [4].

Despite growing recognition of health disparities among minority populations, existing oncology literature has largely overlooked the religion-specific barriers that Muslim patients face in cancer care. While prior studies have examined screening rates among Muslim immigrant women [5-11] and general cultural competence frameworks [12,13], there remains a gap in the integration of these findings into a cohesive, oncology-focused model that addresses the full spectrum of care, from screening to end-of-life. Multiple factors contribute to delayed cancer diagnosis and treatment among Muslim patients, including fatalism, modesty-related avoidance of screening, language barriers, limited health literacy, and mistrust of healthcare systems that lack cultural awareness [5,6,14]. For the purposes of this review, cultural competence refers to the ability of healthcare providers and systems to deliver care that meets the social, cultural, and linguistic needs of patients. Islamic healthcare values encompass principles such as modesty (haya), adherence to halal dietary guidelines, integration of spiritual practices into daily life, and the role of faith in medical decision-making. Faith-based decision making refers to the process by which patients use their religious beliefs to guide choices about diagnosis, treatment, and end-of-life care. Guided by transcultural nursing theory [15], the Crescent of Care Model [16], and principles of cultural humility, this paper explores care models that integrate cultural sensitivity with clinical practice, examines barriers to healthcare access for Muslims, and proposes practical solutions to enhance patient outcomes in oncology settings. A de-identified patient case will also be presented, using a pseudonym for the patient's name.

Specifically, this narrative review aims to (1) examine the intersection of Islamic faith, cultural practices, and oncology care delivery in the United States; (2) identify specific barriers to healthcare utilization among Muslim cancer patients across intrapersonal, interpersonal, institutional, and community levels; and (3) propose practical, evidence-based recommendations for providers to deliver culturally competent oncology care. A narrative, case-based approach was selected because the existing literature on Muslim patients in oncology is heterogeneous in study design, population, and outcomes, making formal systematic synthesis impractical. The case-based format allows integration of diverse evidence streams - clinical, cultural, ethical, and spiritual - into a cohesive and clinically applicable framework.

Literature search strategy

A literature search was conducted using PubMed, Google Scholar, and CINAHL databases. Search terms included combinations of "Muslim patients," "Islamic healthcare," "cultural competence," "oncology," "cancer screening barriers," "modesty in healthcare," "spiritual care," "halal dietary restrictions," "end-of-life care," and "health disparities." The search focused primarily on English-language publications, with emphasis on studies from the past 10 years, though seminal earlier works were included when they remained foundational to the field. Studies were selected based on their relevance to the intersection of Islamic faith, cultural practices, and cancer care delivery. As a narrative review, formal inclusion and exclusion criteria, risk of bias assessment, and systematic synthesis methods such as meta-analysis were not applied; rather, literature was selected to provide a comprehensive and representative overview of the topic. This approach is consistent with the aims of a narrative review, which seeks to synthesize and contextualize existing knowledge rather than quantify effect sizes [5,17].

Not one-size-fits-all: religiosity among U.S. Muslims

**Figure 1:**
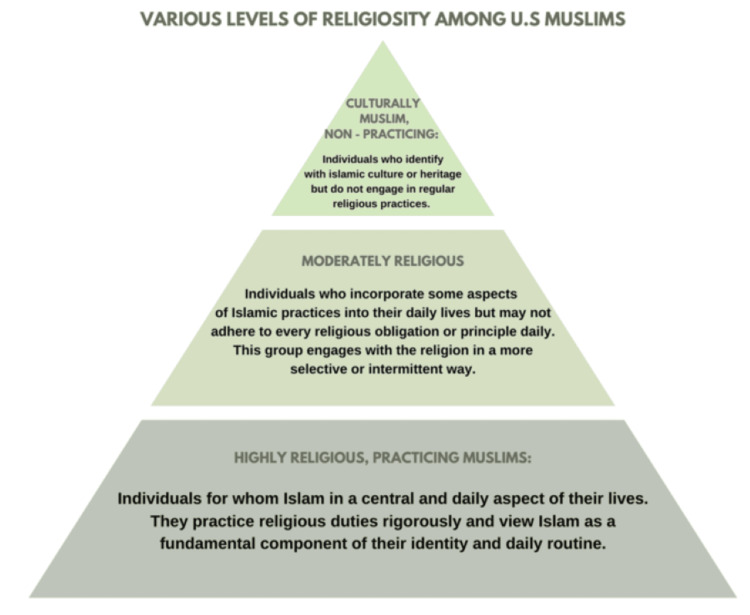
Variations in Religious Practice Among American Muslims This image was created by the authors using Microsoft PowerPoint (Microsoft Corporation, Redmond, WA, USA).

Although religion plays an important role for most American Muslims, there is diversity in how strongly Muslims value religious beliefs. A study in 2017 showed that, on average, 65% of Muslims in the United States stated that religion is particularly important to them [18]. This suggests that a significant proportion of Muslim patients encountered in clinical settings will place significant value on fundamental Islamic principles (Figure 1).

Layers of identity: religion, culture, and the human side of medicine

**Figure 2:**
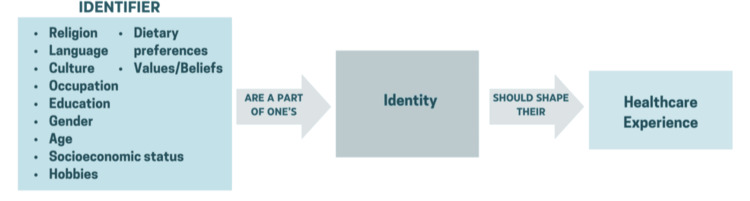
Patient Identifiers and How They Shape the Healthcare Experience This image was created by the authors using Microsoft PowerPoint (Microsoft Corporation, Redmond, WA, USA).

Identity is a broad concept encompassing religion, language, occupation, culture, hobbies, and education, among others. Specifically, religious identity can be very important to the patient. Clinicians should explore whether a patient’s identity is primarily cultural, spiritual, or both, and consider how their beliefs may influence their acceptance or refusal of certain treatment options. Practicing Muslims, for example, may interpret their health and treatment choices through their spiritual beliefs, which may differ from the provider’s perspective. When religious identity is overlooked, patients may struggle to express the importance of their faith in medical decision-making, potentially feeling apprehensive about whether the provider will understand their viewpoint. There is also the concept of intersectional identities, which plays a crucial role in patient care, as shown in Figure 2.

**Figure 3:**
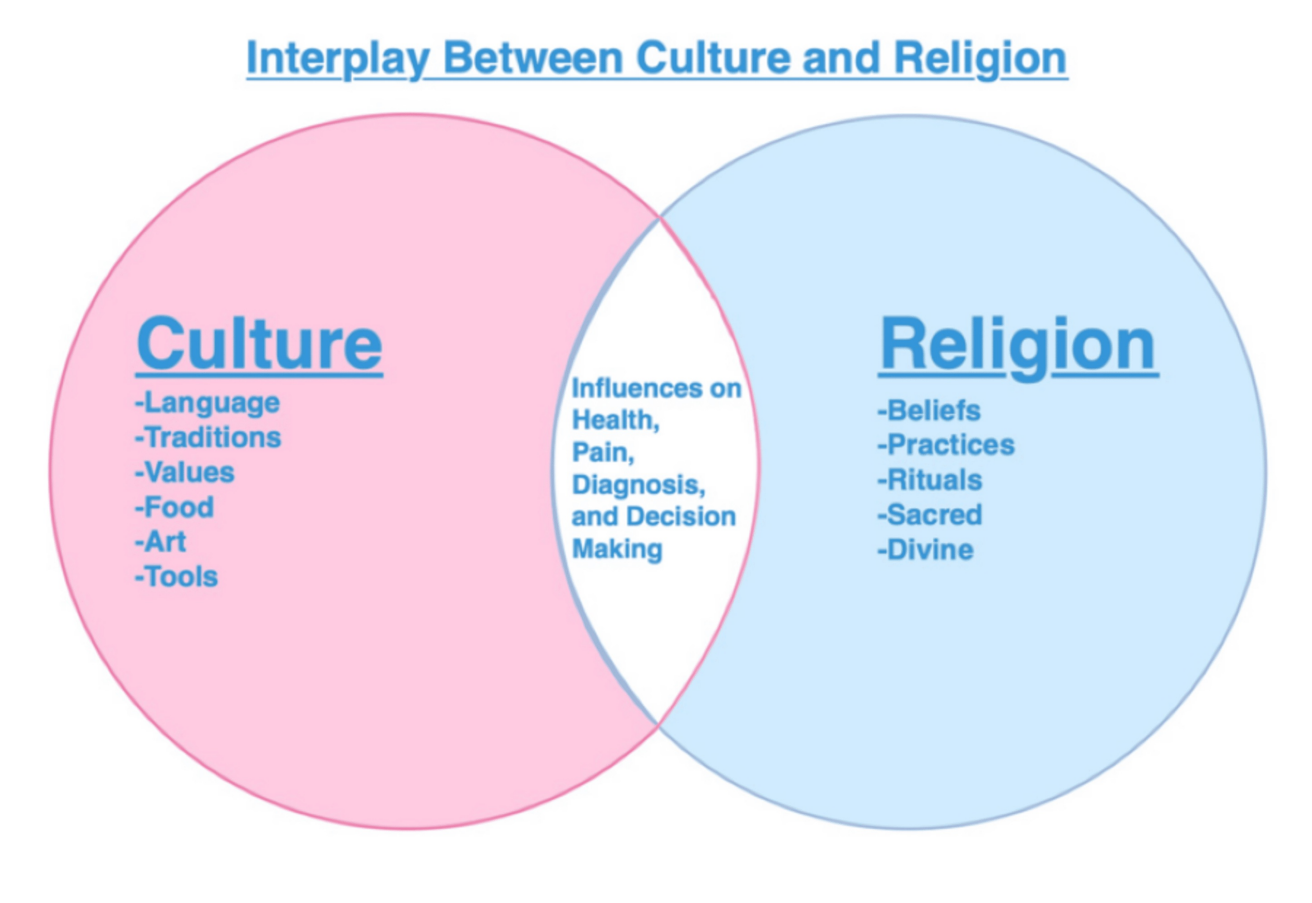
Interplay Between Culture and Religion This image was created by the authors using Microsoft PowerPoint (Microsoft Corporation, Redmond, WA, USA).

For example, a Muslim patient may also identify with other dimensions such as race, age, geographic background, or socioeconomic status. This intersectionality can increase a patient’s vulnerability, especially when the patient has multiple marginalized identities. Culture and religion are intertwined, each influencing how individuals perceive health, disease, pain, and medical decisions. Healthcare providers must be prepared to treat patients from diverse cultural and religious backgrounds, as these factors shape patients’ experiences and decisions [5,19-21]. Figure 3 illustrates the interconnected relationship between culture and religion.

Cultural competence as a catalyst for better care

Culturally competent care improves patient outcomes, not only for Muslims but for patients of all backgrounds [12,13]. This improvement stems from providers understanding their patients' beliefs, enabling care that is both clinically appropriate and culturally respectful, leading to better health behaviors and greater treatment acceptance by patients. Additionally, when providers try to understand a patient's cultural and religious background, it builds trust and encourages open communication. This transparency enables providers to offer highly personalized guidance, helping patients make informed decisions that align with their beliefs. Studies have shown that when a patient's spiritual needs are overlooked or treated as taboo, patients tend to rate the quality of care and their overall satisfaction lower [12]. Demonstrating genuine respect and willingness to accommodate patients’ beliefs fosters a more collaborative approach to care. Engaging the patient in discussions about the risks and benefits can foster trust and provide reassurance for both the patient and their family.

Ethical and cultural dimensions of whole-person care in diverse populations

Providers in the U.S. are trained in an individualistic model, fostering direct patient-provider communication, autonomy, and individual responsibility for healthcare decisions [22]. Although this may provide adequate care for many patients, some patients from diverse cultural backgrounds may benefit greatly from a more collectivist cultural model emphasizing group well-being, whether family or community, over the individual alone [16,23].

A key example is the Crescent of Care Model, which integrates spiritual, cultural, psychosocial, interpersonal, and clinical care to treat a patient holistically [16]. This model emphasizes that family is a crucial support system, incorporating religious accommodations, cultural considerations such as modesty and traditional medicine, family dynamics, strong patient-provider relationships, and high-quality medical treatment.

**Figure 4:**
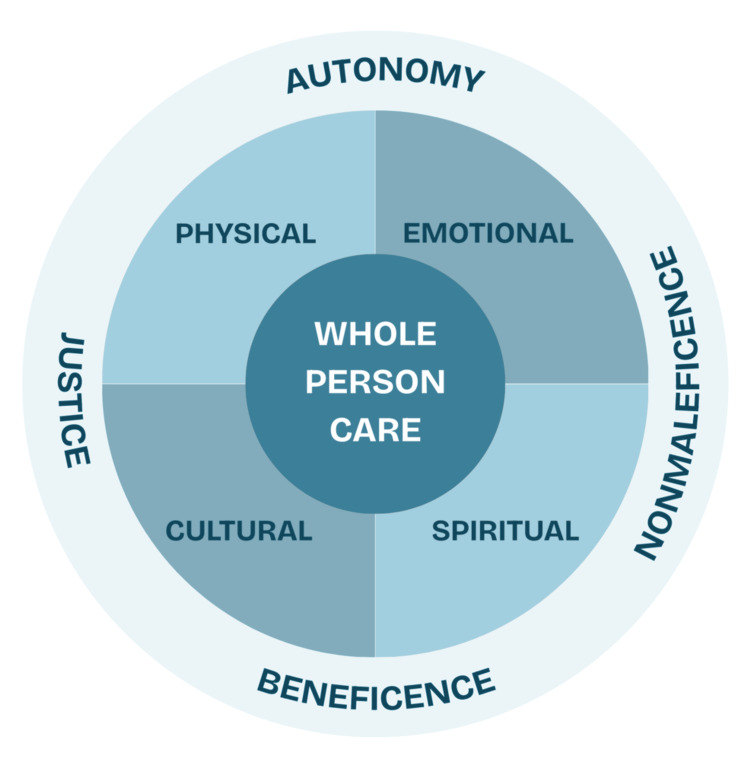
Whole Person Care and Principles of Clinical Ethics This image was created by the authors using Microsoft PowerPoint (Microsoft Corporation, Redmond, WA, USA).

When applying whole-person care, the principal clinical ethics - autonomy (respect for the patient’s right to self-determination), beneficence (obligation to act in the best interest of the patient), non-maleficence (duty to do no harm), and justice (fairness in distribution of resources and treatments) - must serve as foundations for guiding clinical decision-making and ensuring patient-centered care [24]. Figure 4 portrays the interaction between whole-person care and the principles of clinical ethics.

This article is presented as a narrative review with a case-based illustrative example intended to highlight commonly reported cultural and clinical considerations in the care of Muslim oncology patients. Relevant literature was identified through general searches of biomedical databases, including PubMed and Google Scholar, using keywords such as “Muslim patients,” “cultural competence,” “Islam and healthcare,” “oncology,” and “spiritual care.” Articles discussing healthcare disparities, cultural and religious considerations, and patient experiences among Muslim populations were prioritized to support the thematic discussion. As the goal of this work was to synthesize key concepts rather than conduct a formal systematic review, structured systematic review methodologies, such as predefined screening protocols, PRISMA flow diagrams, and formal risk-of-bias assessments, were not applied.

## Review

Cases and discussion

Barriers to Healthcare Utilization: Cultural, Communication, and Systemic Factors

Case: Fatima, a 47-year-old Syrian refugee, arrived in the U.S. in 2016. She wears a Hijab and works as an interior designer. After her divorce in 2018, she became her daughters' primary caretaker. In 2021, she found a breast lump but ignored it, believing, “By the will of God, this will go away.”

Discussion: Western healthcare models often fall short in addressing the specific needs of Muslim patients due to the lack of religious and cultural awareness. Many Muslim patients attribute their emotional distress to spiritual causes and prefer their providers to integrate spirituality into their treatment plans [25]. Neglecting this aspect can alienate patients and foster mistrust, leading them to seek guidance from religious leaders, like Imams, instead of healthcare professionals [26]. This contributes to lower participation rates in healthcare and cancer screenings among Muslim patients, resulting in health disparities and poorer outcomes [6].

For example, studies examining cancer screening behaviors among refugee and immigrant populations have reported lower participation rates in some Muslim-majority groups. In a study of Afghan immigrant women (n = 53), approximately 15% reported receiving a screening mammogram within the past two years of their last checkup. Other studies have reported screening rates of approximately 44% among Iraqi women and 23% among Somali women, compared to higher screening rates reported among other U.S. populations, such as 69% of Black women, 61% of Hispanic women, 60% of American Indian or Alaskan Native women, and 59% of Asian women [6-9]. Furthermore, about 62.2% of American women have had a colonoscopy at some point in their lives, while only 14% of Iraqi women have ever had a colonoscopy [10]. Additionally, 80.7% of American women show a national adherence rate to annual Pap smear screening, while only 51% of Somali women show adherence to Pap smear screening every two to three years [11]. These disparities in cancer screening translate directly into delayed oncology diagnoses, higher rates of advanced-stage presentation, and, ultimately, poorer clinical outcomes for Muslim patients [5,6,14].

Case continued: Over time, the lump grew, with a red spot and increased hardness. Yet Fatima’s main worry was her daughters: “Where would I leave my daughters? I have no family or friends here.” One day, sharp hip pain led her to urgent care, where a fracture was found. Suspecting cancer, she still refused further evaluation, delaying treatment until her mother could arrive five months later. Fatima checked into the hospital the next day.

Discussion: A 2016 study of Arab women in the UAE found that delayed presentation for breast cancer was common, with most patients never having undergone breast screening. Many presented with advanced-stage disease [14]. Another study examined the reasons for delayed care, finding that fear, anxiety, and hesitation were the most common factors contributing to the delay [11]. These factors are likely amplified in countries where most providers are not Muslim. Other reasons for delay included fear of social stigma, lack of knowledge about where to seek care, and discomfort with male doctors [11].

Case continued: At the hospital, Fatima initially refused testing and biopsies. However, meeting a hijab-wearing Muslim female oncologist created an instant connection. Despite Fatima’s fluency in English, the shared cultural and religious background significantly increased her comfort and willingness to engage in care.

Discussion: The barriers faced by Fatima and many Muslims in the U.S. can be categorized into various layers that healthcare providers should strive to understand and address. Table 1 depicts the various barriers that Muslims may face in healthcare, categorizing them into intrapersonal, interpersonal, institutional, and community barriers.

**Table 1:** Muslim Barriers to Healthcare

Level of Barrier	Specific Barrier	Description
Intrapersonal Barriers	Fatalism	Fear of pain from testing and of the result
	Health literacy	Lack of knowledge and awareness about health issues
	Language barriers	Limited proficiency in the receiving country’s language
	Years of residence and acculturation	Limited adaptation due to shorter duration of stay and unfamiliarity with cultural norms
	Beliefs in modesty	Cultural or religious modesty may, for example, decrease participation in breast cancer screening
Interpersonal Barriers	Doctor’s behavior	Doctors are sometimes described as being insensitive or lacking empathy
	Gender preference	A significant factor affecting screening practices among certain cultural groups, such as Arab Muslim women
	Power imbalance between the patient and the clinician	The authority gap, especially across cultural, linguistic, or religious lines, may discourage patients from expressing concerns or participating in decision-making
Institutional Barriers	Discrimination, lack of cultural competency, and weak ties with faith-based organizations	These contribute to feelings of exclusion and mistrust in the healthcare system
Community Barriers	Lack of understanding of the healthcare system	Patients may not know how or where to seek medical help within the receiving country's system
	Lack of transportation or financial resources	Physical and economic inaccessibility hinder access to care
	Family and childcare commitments	Responsibilities at home may prevent individuals - especially women - from attending appointments or undergoing screening
	Shame and taboos	Cultural stigma surrounding illnesses like cancer may discourage open discussion or timely health-seeking behavior

It is important to note that some of these barriers, such as language, are more relevant to non-American or immigrant Muslims and are less of a problem for Muslims who are born or have been acculturated in the United States.

Religious Perspectives on Illness and Their Clinical Implications

Case continued: Fatima gently asked her family to go downstairs for tea, signaling her desire for privacy. Alone with the oncologist, she confided her fear that her illness was punishment for past sins. The doctor patiently listened and spent over an hour addressing Fatima's concerns before carefully explaining the diagnosis of metastatic cancer. Fatima was relieved and surprised to hear that treatment could significantly improve both her survival and quality of life. She asked the doctor to keep her diagnosis confidential.

Discussion: Understanding a patient’s perception of their illness is essential for improving healthcare effectiveness, adherence, and outcomes. Patients’ beliefs about their illness generally fall into four models [23]: (i) medical model - views illness through a biological lens, attributing disease to genetics, hormones, or risk factors; (ii) spiritual/religious model - connects illness to divine will or spiritual shortcomings; (iii) social model - attributes illness to environmental or psychological stressors such as grief or trauma; and (iv) supernatural model - believes illness stems from external mystical forces, such as black magic or the evil eye.

Cultural and religious beliefs play a large role in how Muslim patients understand health and healing. Acknowledging these perspectives fosters respectful and effective healthcare. Table 2 highlights key Islamic beliefs and their relevance to clinical practice. Primary Islamic texts, including translations of the Quran [27] and classical hadith compilations [28], are cited in this review to provide the doctrinal foundations for clinical recommendations, as healthcare providers benefit from understanding the scriptural basis of patients' health-related beliefs and decisions.

**Table 2:** Key Islamic Beliefs and Their Clinical Significance

Belief or Action	Religious Significance to Patients	Clinical Significance to Providers	Reference
Coping With Disease	The Qur’an (the holy book of the Islamic faith) also teaches that God has foreordained all of life's events, and it is God who gives life and causes death. When faced with any trial in life, Muslims are encouraged to face it with patience and prayer.	Discussions of treatment, prognosis, and end-of-life decisions should be sensitive to the understanding that life and death are ultimately in God’s hands. Furthermore, providers should also be aware that Muslims will potentially seek spiritual healing through prayer.	[27]
Visiting the Sick	Inpatient Muslim patients often will have numerous visitors during their stay, as visiting the sick is both a religious and cultural obligation for family members.	This means providers should be prepared for a potentially high volume of visitors. Clear communication with the family about hospital policies is important for maintaining a healthy environment for all patients.	[29]
Health	Health in Islam is a balance of physical, psychological, social, and spiritual well-being.	Providers must adopt a holistic approach when treating Muslim patients, recognizing that their health encompasses more than just physical symptoms or pain.	[30]
Illness	Most Muslims view their illness as a test from God or a part of God’s divine plan. It is believed that through enduring illness, sins are atoned for, ultimately leading to an opportunity for spiritual renewal and reward.	For providers, understanding this will help build trust and rapport, as patients feel their beliefs and values are respected and validated.	[31]
Seeking Treatment	Muslims are urged to seek treatment as the Prophet of Islam said, “O servants of Allah, seek treatment, for Allah has not sent down any illness without sending down its treatment.”	Providers should recognize that Muslims may view their recovery as a religious duty and promote adherence to medical plans and treatments.	[28]
Harm and Prevention in Healthcare	Regarding prevention, anything in Islam that has the potential to cause harm to the body or mind would be considered prohibitive in Islam. This would not include side effects of medications given for a true medical problem.	Health education for Muslim patients on primary prevention should be closely aligned with Islamic teachings that emphasize health promotion and the avoidance of harm.	[15,31]

Integrating Spiritual Care Into Treatment Strategy

Case continued: During bedside rounds, Fatima was often found in bed reciting the Holy Quran. She shared her struggles with maintaining prayers, which caused her anxiety and depression. During a discussion about her treatment, the Muslim physician gently said, “May Allah grant you health and ease your journey.” Fatima smiled, held the physician’s hand, and asked, “Amen, will you pray for me?” The physician warmly replied, “Of course I will.”

Discussion: Spiritual care involves respecting and incorporating a patient’s values and beliefs into their healthcare experience to help alleviate distressing situations [32]. This can include integrating practices such as prayer, meditation, consultation with religious leaders, engagement with holy texts, music, or support from family and friends into the treatment plan. Religion and spirituality are widely recognized as key resources for coping, providing comfort and strength. While healthcare primarily focuses on physical health and treatment - often at the expense of spiritual care - research shows that patients experiencing higher levels of spiritual pain, such as existential distress or spiritual crises, tend to suffer more physically and emotionally than those with little or no spiritual pain [32]. For healthcare providers, it is crucial to acknowledge and support the spiritual practices of Muslim patients, regardless of personal beliefs. For instance, if a Muslim patient diagnosed with a terminal illness wishes to include an Imam in their care to recite Quranic verses in addition to their medical treatment, culturally competent care requires the provider to respect and facilitate this practice. Ignoring or dismissing the spiritual aspect of care can leave patients feeling uncomfortable and undervalued. For Muslim patients, spiritual care may encompass various practices, as outlined in Table 3.

**Table 3:** Islamic Practices and How to Facilitate Them

Islamic Practice	Definition	How to Facilitate the Practice
Prayer	Muslims pray five times a day as it is a fundamental aspect of their daily routine.	Adjusting treatment schedules. Notifying the patient of the timing of medical procedures so that they can plan their prayers accordingly. Providing a quiet space where a patient can concentrate while in the act of prayer.
Wudu	Muslims pray five times a day, requiring ablution before prayer. which involves washing the face, hands, arms, feet, and head.	Understand challenges encountered with IVs or dressings. Allow for dry ablution if water use is not indicated. Consult the Muslim chaplain if needed.
Consulting Faith Leaders	Speaking with a faith leader provides spiritual comfort, reassurance, and a sense of connection to their faith, which can be crucial in navigating the emotional and psychological aspects of illness. Faith leaders can offer prayers, spiritual advice, and interpretations of religious teachings that help patients make sense of their experiences and find peace with their circumstances.	Involving a hospital Muslim chaplain can assist in the Muslim patient experience by ensuring that the patient’s spiritual needs are being met adequately.
Religious Supplications	During times of illness, Muslims engage in the reading and recitation of the Quran, along with other religious supplications such as Ruqia (reciting specific verses of the Quran for healing purposes) and Dhikr (repetitive recitation of God’s names and attributes, which can serve as a form of worship and stress relief).	Providers can support these practices by creating a conducive environment. For practices like Ruqia and Dhikr, it’s important to give the patient the space to recite, ensuring they don't feel judged or that their actions are being dismissed as unimportant.
Modesty	Modesty is fundamental in Islam, affecting both dress and interaction between genders. Women sometimes wear Hijab and cover their body head to toe, and men cover from navel to knee.	Avoid unnecessary interaction with the opposite gender. Match provider and patient genders. Provide long gowns. Knock before entering.
Ramadan Practices	Ramadan includes fasting (abstaining from food, water, and sexual activity) from dawn to sunset.	The medically ill are exempt from fasting but many may insist. Consult a Muslim chaplain for guidance. Show empathy for fasting concerns.
Dietary Restrictions	Alcohol and pork; this may extend to food and medicine that contain alcohol and pork products.	Use non-porcine products when possible. Provide halal food or vegetarian/seafood options.
End of Life Care	Death is seen as a communal affair and family involvement is common. Reciting the Shahada before death is important.	Facilitate the presence of family or a chaplain. Attempt to understand and respect end-of-life beliefs and encourage shared decision-making.

In reality, healthcare providers frequently face time constraints, limiting their ability to address a patient's spiritual needs. Furthermore, existing spiritual care programs may not be well-suited to the practical demands of the hospital environment, making it challenging for healthcare providers to implement them effectively. Compounding the issue, many clinicians do not receive training in spiritual care during their medical education, highlighting the need to integrate this crucial aspect into healthcare provider training.

Important Muslim considerations

Modesty

Case continued: During her hospital stay, Fatima needed help showering but hesitated when a male nurse arrived, choosing to postpone it. Later, a male medical student came to examine her breast mass. Though visibly uncomfortable, she did not voice her concerns.

Discussion: There are important Islamic considerations that providers should be aware of when treating Muslim patients. In Islam, modesty is a core principle in daily life, encompassing both dress and interactions between genders. For women, modesty is often expressed through the hijab (headscarf), which covers the hair, along with clothing that covers the entire body except for the face and hands [30]. This concept of modesty also applies to men, who are required to cover their bodies from the navel (umbilical area) to the knee [33]. Modesty further extends to interactions between men and women, with both genders advised to avoid unnecessary contact with the opposite sex [34]. However, Islamic teachings allow exceptions in situations where there is no alternative or during emergencies [27].

It is also crucial to recognize that, as mentioned earlier, there are varying levels of religiosity among Muslims, meaning that expressions of modesty may differ from patient to patient. Therefore, it is essential to respectfully inquire about each patient’s preferences.

Hospitals can help maintain the modesty of their Muslim patients by offering long hospital gowns, knocking on the door before entering a room and allowing time for the patient to dress, posting a sign outside the patient’s room to remind providers that the patient may be wearing a hijab, and accommodating gender preferences for providers. In emergencies, where examinations or procedures may require uncovering parts of the body or interacting with a provider of the opposite gender, Islamic prohibitions on these matters are lifted [27].

Practices During the Month of Ramadan

Case continued: Two months later, Fatima was readmitted with nausea and vomiting, appearing weak and thin as her cancer progressed. With Ramadan approaching, she expressed concern, asking, “Can I still fast during Ramadan?”

Discussion: Another important consideration is the month of Ramadan, the ninth month of the Muslim lunar calendar. During Ramadan, Muslims fast from dawn until sunset, abstaining from food, water, and sexual intercourse. The month also includes congregational prayers, spiritual reflection, and communal activities and is often regarded as the most significant month of the year for Muslims. When a medical condition prevents someone from fasting, it can make them feel excluded or disconnected. While Islam exempts those who are medically ill from fasting, many Muslims may still insist on fasting despite having religious flexibility [27].

In such cases, involving a Muslim chaplain can provide spiritual guidance on fasting. Clinicians should share medical concerns with empathy, as dismissing a patient's desire to fast may harm trust. Even if fasting is not medically advised, acknowledging Ramadan’s significance and addressing concerns with respect fosters a better patient-provider relationship.

Dietary Restrictions

Case continued: While hospitalized, Fatima's caloric intake remained poor as she refused most hospital food. She then developed a pulmonary embolism. Since the facility's heparin was porcine-derived, the clinician opted for an alternative anticoagulant to respect her dietary restrictions.

Discussion: Islam includes specific dietary restrictions, with alcohol and pork products being strictly prohibited, although there are differing opinions on some other issues. These prohibitions may extend to any food or medicine containing these products. Permissible foods are known as halal. This includes fish and meat that have been slaughtered according to Islamic guidelines - this involves invoking the name of God during the slaughter and ensuring the animal is treated humanely. For patients who prefer their meat to be strictly halal, hospitals can accommodate their dietary needs by offering vegetarian or seafood options.

Prayers

Case continued: Fatima prays five times a day. As her condition worsens, she grows fatigued, making it difficult to pray while standing.

Discussion: As previously mentioned, prayer is a core practice in Islam [27]. Understanding its significance and associated rituals is essential for providers caring for Muslim patients. Muslims pray five times daily, facing Mecca, Saudi Arabia. These prayers occur at specific times throughout the day, beginning at dawn and ending at night. Before prayer, Muslims must achieve spiritual purity through ablution, which involves washing the face, hands, arms, feet, and head [27].

Prayer can be challenging in healthcare settings, especially when IV lines or dressings hinder ritual washing. In such cases, dry ablution (tayammum) can be used as an alternative. Thoughtful considerations, such as informing patients of the Qibla direction or adjusting bed orientation when possible, show respect for religious practices. If prayer becomes impractical due to health risks, Islamic teachings permit its suspension [27]. Consulting a Muslim chaplain can help ease patients' concerns and provide spiritual reassurance. 

End-of-Life Care

Case continued: Eighteen months later, Fatima was readmitted, weak, cachectic, and jaundiced, unable to stand unsupported. Realizing death was near, she held the oncologist’s hands, crying silently. She feared dying alone in the hospital, suffering, and being too medicated to recite her Shahada. She also asked about the care of her body if she passed away in the hospital.

Discussion: Islamic beliefs emphasize preparing for death as an inevitable event that cannot be delayed [29]. Dying is viewed as a communal experience, with family members holding a spiritual duty to care and pray for their sick and dying relatives. Many Muslims seek to recite the Shahada (the Islamic declaration of faith) before passing away. Facilitating this practice through chaplains, family involvement, or hospital staff can provide spiritual comfort [17].

Despite the benefits of palliative and hospice care, Muslim patients’ specific needs are often not met, leading to lower hospice enrollment and higher hospital deaths [35]. Many Muslims are unfamiliar with or hold misconceptions about these services, fearing conflicts with their faith [35]. While most are open to receiving pain medication, concerns about spiritual interference and beliefs in redemptive suffering may lead some patients to reject symptom-focused palliative care.

Clinical recommendations: improving the healthcare experience for Muslim patients

Improving Health Literacy and Access

Fatima’s initial hesitation to seek medical attention, along with statements like “By the will of God this will go away,” underscores the need for health education that is tailored to diverse cultural and religious beliefs. This could involve creating simple educational materials that explain common disease symptoms and treatment options in a way that aligns with faith and beliefs. Additionally, partnerships between hospitals and local mosques or community centers can play a crucial role in spreading healthcare information and emphasizing the importance of timely medical care. Such collaborations can also enhance the cultural competency of healthcare providers by deepening their understanding of Islamic traditions through firsthand exposure to these practices. This was exemplified in Fatima’s positive interaction with the female oncologist.

Communication and trust-building strategies

Enhancing Provider-Patient Interactions

Healthcare providers should adopt the "Ask, Don't Judge" approach to foster a safe environment where Muslim patients feel comfortable discussing their concerns. Providing access to spiritual care resources, such as a Muslim chaplain or counseling services, can offer additional support and reassurance. Gender-sensitive care is equally important, as seen in Fatima’s reluctance to interact with male healthcare providers. Ensuring that both female and male staff are available for personal procedures is essential for delivering compassionate and respectful care to Muslim patients.

Addressing Barriers and Providing Support

Fatima’s isolation and concern about her daughters address the need for a comprehensive support system for both Muslims and non-Muslims. This can include services such as counseling and community support groups with others in similar situations. Childcare arrangements could also have alleviated some burdens in receiving timely medical care. Fatima’s subpar caloric intake points toward nutritional and dietary considerations. Hospitals should strive to offer halal options for Muslim patients. Lastly, Fatima’s fears about dying alone, her body’s care, and her concern to perform her Islamic rites before death necessitate a compassionate end-of-life care approach. Religious and cultural preferences should be addressed and respected.

## Conclusions

Given the large and increasing Muslim population in the United States, healthcare providers are likely to care for Muslim patients whose faith and beliefs profoundly influence their healthcare experiences. Providers must be aware of and sensitive to barriers that may hinder healthcare utilization, including language challenges, family dynamics in decision-making, and religious considerations. Adopting an “Ask, Don’t Judge” approach fosters open communication, trust, and respect for patients’ cultural and religious values, ultimately enhancing patient-centered oncology care. Practical steps for healthcare institutions include implementing cultural competency training programs for oncology staff; establishing relationships with local Muslim community organizations and chaplaincy services; modifying electronic health records to capture patients' religious and cultural preferences; ensuring availability of gender-concordant providers and halal dietary options; and developing palliative care protocols that accommodate Islamic end-of-life practices.

Future research should focus on prospective studies evaluating the impact of these culturally tailored interventions on measurable oncology outcomes, such as screening adherence, time to diagnosis, treatment completion rates, and patient-reported satisfaction among Muslim patient populations. This article is a narrative review and does not present original clinical data. The case described is a fictionalized, illustrative example intended to highlight commonly reported challenges in the literature and may not be generalizable to all Muslim patients or clinical settings, given variations in cultural practices, levels of religiosity, and healthcare systems. Lastly, it is essential to remember that, in emergencies or situations where avoiding religious prohibitions is difficult, saving a life always takes precedence over religious restrictions.
